# Highlights from the Brain Conference 2025

**DOI:** 10.1093/braincomms/fcaf143

**Published:** 2025-05-02

**Authors:** Manuela Marescotti

**Affiliations:** Edinburgh, UK

## Abstract

Our Scientific Editor reports on the highlights of the Brain Conference 2025, and announces the winner of our Early Career Researcher Award for a paper published in the journal in 2024.

## Graphical Abstract

**Graphical Abstract:**
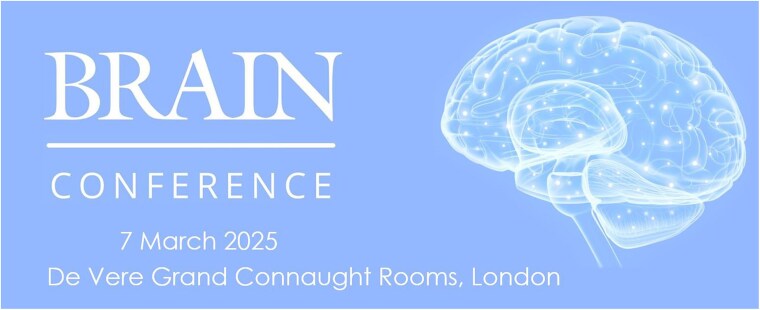

Welcome to Volume 7 Issue 3 of *Brain Communications*. In this editorial, we share with our readers the highlights of the Brain Conference 2025 (https://conference.guarantorsofbrain.org/), and announce the winner of the third *Brain Communications* Early Career Researcher (ECR) Award for a paper published in the journal in 2024.

This year, the Brain Conference was held in London on 7 March, and I had the opportunity to be one of the organizers of this event. Looking at the genesis of this conference, I witnessed the organizers’ passion and effort in creating a programme dense with great science and, at the same time, a smooth schedule for the attendees to follow. This further confirms that the Brain Conference, now in its fifth edition, is an annual appointment that neuroscientists based in the UK should attend.

The one-day conference was structured into different sessions: a plenary talk, topic-related talks (Movement Disorder, Cognition and Dementia, and Inflammation in Neuroscience, with three talks related to each topic), ‘Data Blitz’ talks and a poster session, not necessarily in this order. The speakers for the plenary lecture and the talk sessions were invited by the organizers, based on their outstanding contributions to their research fields. On the other hand, the speakers of the Data Blitzes session were more junior scientists: the *Brain Communications* ECR Award 2024 winner and poster presenters, whose abstracts stood out during the abstract review stage. In addition, the Data Blitz speakers also had the opportunity to present their research studies during the poster session over the lunch break.

Given the primary commitment of the Guarantors of Brain to education, the conference started with the plenary lecture by Professor Darren Monckton (University of Glasgow) about current advances on the role of unstable DNA in Huntington’s disease. Professor Monckton discussed insights from genetic studies done to identify the molecular predictors of this disease, which appear to be CAG repeats more than polyglutamine. In addition, Professor Monckton focused on research on new effective therapies aimed at identifying the ideal treatment window for starting administration and the mechanism required to make sure that the affected cells do not reach >150 repeats, which looks to be the critical threshold for number of repeats.^1^

Speakers in the Movement Disorder session illustrated three different research approaches for studying Parkinson’s disease. First, Professor Huiling Tan (University of Oxford) drew the audience’s attention to the regulation of beta-oscillations and gamma-oscillation within deep brain stimulation as a treatment for Parkinson’s disease, as beta-oscillations appear to be associated with untreated Parkinson’s disease patients, while gamma-oscillations seem to play a role in reinvigorating movement.^2^ Second, Professor Sanjay Manohar (University of Oxford) explored the four aspects of motivation (reward-driven, overcoming effort, energising and call-directed) and how they are disrupted in Parkinson’s disease. Third, Dr Mie Rizig (University College London) led us through the advantages of ancestral genomics to identify the aetiology of diseases, accelerate medical discoveries and ensure equal healthcare access for everyone regardless of their ethnicity and be cost-effective.

In the Cognition and Dementia session, the speakers reported evidence that not only neurons but also other systems in the body, or non-neuron cells within the brain, have a role in neurodegenerative diseases. Professor John Cryan (University College Cork) discussed the interaction between the gut and brain and data from his research group about the gut microbiome being a target for studying Alzheimer’s disease. Of note, he observed that transplanting gut microbiota from Alzheimer’s patients to young, healthy mice also led to the transfer of memory impairment in the recipient animals.^3^ Then, Professor David Atwell (University College London) illustrated the connection between dementia and the vascular system, in particular, the role of pericytes in the reduction in blood flow to the brain in Alzheimer’s patients. Interestingly, this pathway was shown in mice to be a promising target for increasing the blood flow in Alzheimer’s disease with oral administration of nimodipine.^4^ Lastly, our Editor-in-chief, Professor Tara Spires-Jones (University of Edinburgh) showed evidence of the involvement of synapse–astrocyte interaction in Alzheimer’s disease pathogenesis, indicating that they may play an even stronger role in this disease than microglia, in particular, reporting that astrocytes engulf pre- and post-synapses around Alzheimer’s disease plaques.^5^

The Data Blitz talks (5 min each) were shorter than the talks in the other sessions but still very insightful, covering several research methodologies. During the Data Blitzes session, the *Brain Communications* ECR Award 2024 was presented to Angelika Zarkali (PhD student, University College London) as the first author of an outstanding paper published in *Brain Communications* in 2024, entitled ‘Neuroimaging and plasma evidence of early white matter loss in Parkinson’s disease with poor outcomes.’^6^ Nominations for the award were invited in January 2025, as announced in our first editorial of volume 7.^7^ There were two stages to the selection process: first, nominations were invited from the scientific community, for a ‘favourite’ paper with an ECR first author, and a second, the *Brain Communications* editors were invited to choose the winner from the papers selected during the first stage. Ms Zarkali gave a talk about her winning article during this session. Zarkali *et al.*^6^ used multimodal imaging and plasma markers analysis in Parkinson’s disease patients with a poor clinical course. Their work showed the presence of white matter macrostructural changes and increased neurofilament light chain levels. We would like to take this opportunity to thank all those who nominated papers for this award, the nominees, and our editors for their contribution to this initiative, and to congratulate Ms Angeliki Zarkali once again for her award.

The Inflammation in Neuroscience session covered the relationship between the immune system and the brain through different lines of research. Professor Neil Harrison (Cardiff University) presented evidence of the cross-talk between the immune system and the brain having an impact on mood, reward and punishment processing in depression, and lastly, memory. Then, Dr Maura Malpetti (University of Cambridge) reported evidence of the involvement of inflammation in fronto-temporal dementia and also that screening inflammation markers in patients’ blood samples can be informative about this disease and other neurodegenerations. Last, Professor Angela Vincent (University of Oxford) shared her thoughts about antibody-mediated brain disorders based on her long commitment to this research area.

The Brain Conference concluded with final remarks from the Professor Mary Reilly on behalf of the Guarantors of Brain, and the Poster Prize winner presentation to Federica Genovese (PhD student, University of Edinburgh) for her poster about her work to identify the molecular causes of the pre-natal manifestations of spinal muscular atrophy.

The cover of this issue shows a hippocampal neuron from a dissociated mouse neuron culture expressing the fluorescent protein TdTomato, image courtesy of Dr Francesco Gobbo, Edinburgh University.

## References

[1] Handsaker RE, Kashin S, Reed NM, et al Long somatic DNA-repeat expansion drives neurodegeneration in Huntington disease. Cell. 2025;188(3):623–639.e19. 39824182 10.1016/j.cell.2024.11.038 PMC11822645

[2] He S, Baig F, Merla A, et al Beta-triggered adaptive deep brain stimulation during reaching movement in Parkinson’s disease. Brain. 2023;146(12):5015–5030. 37433037 10.1093/brain/awad233 PMC10690014

[3] Grabrucker S, Marizzoni M, Silajžić E, et al Microbiota from Alzheimer’s patients induce deficits in cognition and hippocampal neurogenesis. Brain. 2023;146(12):4916–4934. 37849234 10.1093/brain/awad303 PMC10689930

[4] Hernández JCC, Bracko O, Kersbergen CJ, et al Neutrophil adhesion in brain capillaries reduces cortical blood flow and impairs memory function in Alzheimer’s disease mouse models. Nat Neurosci. 2019;22(3):413–420. 30742116 10.1038/s41593-018-0329-4 PMC6508667

[5] Tzioras M, Daniels MJD, Davies C, et al Human astrocytes and microglia show augmented ingestion of synapses in Alzheimer’s disease via MFG-E8. Cell Rep Med. 2023;4(9):101175. 37652017 10.1016/j.xcrm.2023.101175 PMC10518633

[6] Zarkali A, Hannaway N, Mccolgan P, et al Neuroimaging and plasma evidence of early white matter loss in Parkinson’s disease with poor outcomes. Brain Commun. 2024;6(3):fcae130. 38715714 10.1093/braincomms/fcae130 PMC11073930

[7] Spires-Jones TL . Data management (and sharing) in neuroscience: Balancing possible, practical and perfect solutions. Brain Commun. 2024;7(1):fcae450. 39723108 10.1093/braincomms/fcae450 PMC11668176

